# Held Up in Traffic—Defects in the Trafficking Machinery in Charcot-Marie-Tooth Disease

**DOI:** 10.3389/fnmol.2021.695294

**Published:** 2021-08-16

**Authors:** Ronja Markworth, Mathias Bähr, Katja Burk

**Affiliations:** ^1^Department of Neurology, University Medical Center Göttingen, Göttingen, Germany; ^2^Center for Biostructural Imaging of Neurodegeneration, Göttingen, Germany

**Keywords:** Charcot-Marie-Tooth, trafficking, signaling, transport, endosomal system

## Abstract

Charcot-Marie-Tooth disease (CMT), also known as motor and sensory neuropathy, describes a clinically and genetically heterogenous group of disorders affecting the peripheral nervous system. CMT typically arises in early adulthood and is manifested by progressive loss of motor and sensory functions; however, the mechanisms leading to the pathogenesis are not fully understood. In this review, we discuss disrupted intracellular transport as a common denominator in the pathogenesis of different CMT subtypes. Intracellular transport via the endosomal system is essential for the delivery of lipids, proteins, and organelles bidirectionally to synapses and the soma. As neurons of the peripheral nervous system are amongst the longest neurons in the human body, they are particularly susceptible to damage of the intracellular transport system, leading to a loss in axonal integrity and neuronal death. Interestingly, defects in intracellular transport, both in neurons and Schwann cells, have been found to provoke disease. This review explains the mechanisms of trafficking and subsequently summarizes and discusses the latest findings on how defects in trafficking lead to CMT. A deeper understanding of intracellular trafficking defects in CMT will expand our understanding of CMT pathogenesis and will provide novel approaches for therapeutic treatments.

## Introduction

Charcot-Marie-Tooth disorder (CMT) is a group of hereditary peripheral neuropathies leading to loss of sensation and fine motor control in the extremities with a prevalence of over 1:2,500. Over 100 genes are identified to cause CMT (Timmerman et al., 2014; Rudnik-Schöneborn et al., 2020). CMT is diagnosed and categorized into several subtypes based on clinical presentation including a loss of sensation and fine motor control in the extremities, nerve conduction velocity, neuropathological findings, as well as mode of inheritance and genes involved (Bird, 2020). Classically, CMT1 is a demyelinating neuropathy, CMT2 is an axonal neuropathy, and dominant-intermediate CMT (DI-CMT) is an intermediate type showing both demyelinating and axonal pathologies. Since many more genes causing CMT were discovered showing overlaps of the phenotypes, a gene-based classification of inherited neuropathies has been proposed. Since so far this classification is not established, we stick to the classification by genes with the corresponding alphanumeric classification according to OMIM (Magy et al., 2018).

The observation that so many different mutations lead to a very similar phenotype has stirred the idea for a common pathomechanism and led to a search of a molecular pathway that covers many of the proteins recorded in CMT. Another interesting aspect of CMT is the peripheral nerve specificity, regardless of the ubiquitous expression of many of the proteins associated with CMT. Peripheral neurons stand out due to their length and polarity. Both sensory and motor neurons of the peripheral nervous system (PNS) can be up to over a meter in length and symptoms often arise earliest in the extremities. This, in combination with the post mitotic state of the neurons, makes these cells particularly susceptible to changes in the intracellular transport machinery.

**FIGURE 1:**
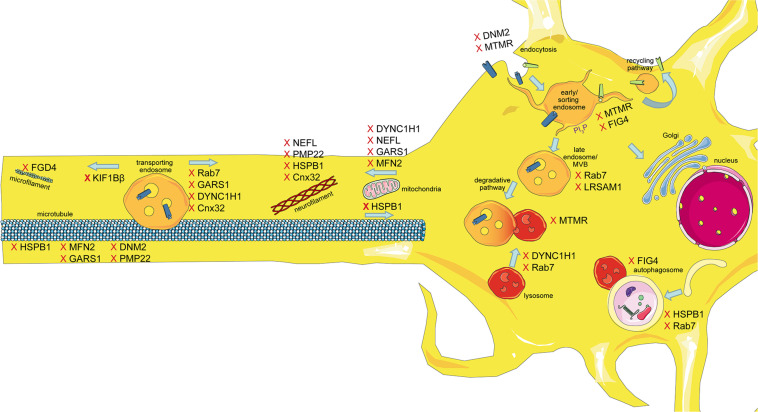
Schematic overview of the intracellular trafficking processes in a neuron of the PNS. Highlighting all the steps where proteins involved in CMT can cause dysregulation. This figure was created using Servier Medical Art templates, which are licensed under a Creative Commons Attribution 3.0 Unported License; https://smart.servier.com.

**FIGURE 2:**
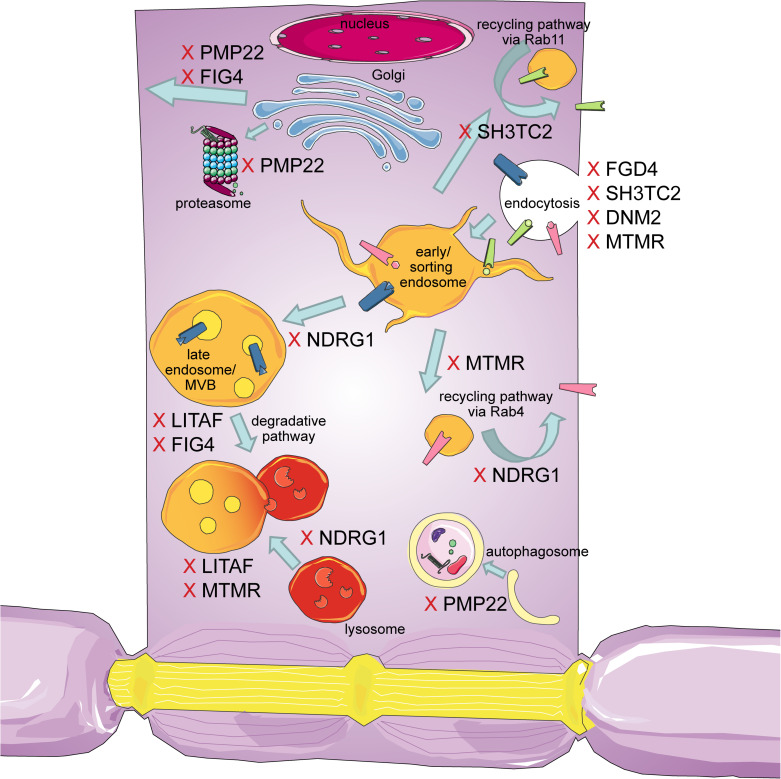
Schematic overview of the intracellular trafficking processes in a myelinating Schwann cell. Highlighting all the steps where proteins involved in CMT can cause dysregulation. This figure was created using Servier Medical Art templates, which are licensed under a Creative Commons Attribution 3.0 Unported License; https://smart.servier.com.

The intracellular transport machinery ranging from endocytosis to protein degradation is a tightly regulated, yet extremely dynamic system that is crucial for energy metabolism, signaling and protein homeostasis. Even though many studies look at trafficking aspects in the different types of CMT, we are lacking studies that look at multiple CMT types at once to benefit from directly comparable results. Here we will give an overview of the different aspects involved in intracellular trafficking (the irony of trying to divide such a dynamic system into clearly defined sections is not lost on us). We briefly mention which CMT-mutations show defects in the respective sections but discuss several CMT disorders, their trafficking defects and similarities between them after presenting the cell biological mechanisms. Our main approach is to link the different pathological mechanisms seen in CMT to find common denominators. An overall summary of how genes causing CMT affect trafficking can be found in Figure 1 (axonal) and Figure 2 (Schwann cell).

## Endocytosis

Arguably, the first step for intracellular transport is endocytosis. The most common and well-studied pathway for the uptake of nutrients, signaling receptors but also ion channels, transporters or pathogens, as well as vesicles is clathrin mediated endocytosis. Upon initiation signal [for many signaling receptors this involves kinase activity and mono-ubiquitination of the intracellular domain (Haglund et al., 2003)] Phosphatidylinositol (PI) 4,5-biphosphate (PI_4_,_5_P_2_) is generated at the plasma membrane, which recruits AP2, an adaptor protein complex. AP2 then recruits clathrin triskella that—with the involvement of several other proteins such as F-BAR domain-containing Fer/Cip4 homology domain-only proteins and Epsins-induce and stabilize membrane curvature and form a clathrin-coated pit, in which cargo gathers. Subsequently, Dynamin is recruited to the neck of the pit and self-polymerizes to induce membrane scission by GTP hydrolysis. The clathrin-coated vesicle pinches off subsequently (reviewed in McMahon and Boucrot, 2011). Upon removal of the clathrin coat, the vesicle is ready to fuse with early endosomes and begins its journey along the intracellular trafficking pathways.

Several genes known to cause CMT have been associated with dysfunctional endocytic processes, namely *MTMR2,13,5, SH3TC2, FGD4, DNM2* (relating to the subtypes CMT4B, CMT4C,CMT4H, and DI-CMTB, respectively), which will be discussed below. Defects in endocytosis have brought consequences on neuronal health for example lack of endocytosis of neurotrophic receptors would eventually affect gene expression of pro survival genes (Scott-Solomon and Kuruvilla, 2018).

From the early endosome, cargo continues its intracellular journey. The early endosome is characterized by the binding of the Rab5 GTPase, a small Rab GTPase that is cytosolic in its inactive GDP bound state. Upon activation by a Guanine nucleotide exchange factor (GEF) Rab5 is membrane tethered and activates several effector proteins, such as early endosome antigen 1 (EEA1; Gorvel et al., 1991; Bucci et al., 1992; Simonsen et al., 1998). The early endosome has a slightly acidic milieu with a pH of 6, which is generated by the vacuolar H-ATPase (Johnson et al., 1993). The membrane contains a high content of PI_3_P and its shape is characterized by tubular extensions from its sorting activity (Jovic et al., 2010). The early endosome is also often referred to as the sorting platform. From there, cargo is sorted into one of three possible pathways. The recycling pathway, which is considered the default pathway, the retrograde pathway back to the trans golgi network (TGN) or to the soma, and lastly the degradative pathway (Schreij et al., 2016).

## Recycling

After the clathrin coat is shed, the early endosome acts as a sorting station where bulk recycling is the default pathway due to probability: The endosome extends tubular domains increasing the amount of membrane in this section. Therefore, more receptors per volume will end up on that tubular domain, which is pinched off and recycled back to the plasma membrane (Maxfield and McGraw, 2004). Due to the acidic lumen of the endosome, many ligands dissociate from their receptors. Consequently, only the receptor is shuttled back to the plasma membrane for another round of signaling. Ligands, however, remain in the endosome for maturation and subsequent degradation. A prime example for this pathway is Transferrin and its receptor (Hopkins and Trowbridge, 1983). Receptors can also be recycled specifically via the so-called retromer complex together with the WASH complex and dynamin 2 (Derivery et al., 2009; Seaman et al., 2013). For example, the β-adrenergic receptor is recycled via a guided retromer sorting nexin (SNx) 27 complex. Where SNx27 recognizes a recycling sequence at the C-terminal of β-adrenergic receptor (Lauffer et al., 2010; Temkin et al., 2011; Seaman, 2012). This recycling mechanism has also been reported for other receptors, using different retromer/SNx combinations (Weeratunga et al., 2020). It is to be expected that many more receptors have specific sorting sequences interacting with different adaptors leading to a highly selective sorting mechanism. Rab4 and Rab11 decorate recycling endosomes undergoing fast and slow recycling, respectively (Jovic et al., 2010). Defects in recycling can either affect re-activation due to decreased surface levels of the receptor or affect downstream signaling from endosomes causing for example growth defects (Pasterkamp and Burk, 2020).

Defects in recycling have been reported for mutations in *DNM2, MTMR2,13,5 SH3TC2*, and *NDRG1*, causing DI-CMT, CMT4B, C and 4D, respectively.

## Endosomal Maturation

As mentioned above, the early endosome undergoes a maturation process, from so-called early endosomes to late endosomes. This maturation is manifested in a Rab switch, where Rab5 activates effectors that are Rab7 GEFs, which, in turn, activate Rab7 (Rink et al., 2005). The luminal content of the endosome further acidifies (pH between 4.5 and 6) and a shift in PI composition (PI_3_P to PI_3_,_5_P_2_) by 1-phosphatidylinositol 3-phosphate 5-kinase (PIKFYVE) is reported (Maxfield and Yamashiro, 1987; Wallroth and Haucke, 2018). Maturation is further manifested by the formation of intra-luminal vesicles (ILVs) by inward budding of the maturing endosomal membrane. These ILVs contain cargos marked for degradation by ubiquitination. At this point, late endosomes are often referred to as multi vesicular bodies (MVBs). Late endosomes/MVBs have also been reported to be involved in retrograde trafficking. The following sections describe the three different pathways that all fall under the category of retrograde transport, namely, retrograde transport for signaling, retrograde transport to the TGN, and retrograde transport for degradation.

## Retrograde Transport for Signaling

While many receptors signal from the plasma membrane, others rely on internalization in signaling endosomes to propagate their signaling cascade. Neurotrophins as well as neurocytokines are retrogradely trafficked, mostly during development. Even though tropomyosin-related kinase A (TrkA) and B (TrkB), the receptors for neurotrophins nerve growth factor (NGF) and brain-derived neurotrophic factor (BDNF), respectively, are mostly studied for their role in neurodevelopment, TrkB has been shown to be import for the maintenance of neurons in the adult neocortex (Xu et al., 2000). In addition, peripheral sympathetic neurons that were treated with an anti-NGF antibody slowly degenerated, indicating that not only TrkB but also TrkA has a role in maintenance of the nervous system (Ruit et al., 1990).

Neurotrophic receptors that were endocytosed after monoubiquitination, have been shown to be transported in early or late endosomes in different model systems (Delcroix et al., 2003; Deinhardt et al., 2006). Recently a study showed that TrkA is transported along the axon of sympathetic neurons in MVBs but signals from small vesicles at the soma (Ye et al., 2018). However, endosomal maturation is a fluent process (e.g., as described during the Rab-switch), making it hard to pinpoint the exact maturation state of endosomes. In addition, static studies without any live-cell imaging, only give very limited insight.

Disruption in retrograde traffic for signaling have been observed in mutations in *RAB7* (CMT2B), *GARS1* (CMT2D), *HSPB1* (CMT2F). Neurotrophic signaling regulating neuronal survival depends on retrograde trafficking (Scott-Solomon and Kuruvilla, 2018).

## Retrograde Transport to the TGN

When retrograde transport is mentioned in non-neuronal cells, it is referring to the transport of cargo from endosomes to the TGN. In this context, the most studied cargo being the cation-independent mannose 6 phosphate receptor (CIMPR) that delivers hydrolases to late endosomes/lysosomes. The retromer complex, a key player in the endosomal sorting machinery, regulates this process. Composed of a vacuolar protein sorting-associated protein (VPS) trimer (VPS26,29,35), termed cargo recognition complex, and a SNx dimer (SNx1, 2 with SNx5 or 6), this protein complex returns the receptor back to the TGN, where it can pick up a fresh round of hydrolases (Seaman, 2004, 2012). SNxs are BAR-domain containing proteins, which drive membrane binding and curvature via their PI_3_P membrane binding domain. The VPS trimer recruits CIMPR into a tubulus, which is stabilized by another protein complex termed WASH complex (Wiskott–Aldrich syndrome protein and SCAR homolog complex). Thoroughly studied in yeast, the exact roles of the VPS trimer and SNxs are still being debated in human cells (Kvainickas et al., 2017; Simonetti et al., 2017). When retrograde vesicles containing CIMPR reach the TGN, they need to dock and fuse for further hydrolase uptake by the receptor. This docking and fusion is orchestrated by Golgi-associated retrograde protein complex (GARP) and Rab29 (Bonifacino and Hierro, 2011; Wang et al., 2014). If this pathway is disrupted no more hydrolases are delivered to lysosomes, leading to a lack in degradation, therefore to an accumulation of neurotoxic waste, a lack of nutrients and triggered apoptosis.

Disruptions of retrograde traffic and accumulations in the TGN were observed for mutations in *GJB1* causing, CMT1X.

## Retrograde Transport for Degradation

As just discussed, retrograde transport of CIMPR is very important for proper lysosomal function, a process essential for protein degradation. Internalization and subsequent degradation of signaling receptors is an important step in signal termination. Polyubiquitinated proteins at the cell surface that are marked for degradation remain in the early endosome (aka no recycling) while this endosome matures into a late endosome. The ubiquitinated cargo is recognized by Hrs of the endosomal sorting complexes required for transport (ESCRT)-0 complex (Raiborg et al., 2002). Interacting with PI_3_P ESCRT-0 recruits ESCRT-I and II, ubiquitinated cargo is concentrated in clathrin coated microdomains (Katzmann et al., 2001; Bache et al., 2003). ESCRT-III recruits membrane curving proteins to generate ILVs (Adell et al., 2014). Once a late endosome contains such ILVs, it is classified as a MVB. The definition of a MVB throughout the literature is quite vague when it comes to marker proteins—MVBs are only definitely characterized structurally in electron microscopy images. Upon ILV generation, the MVB fuses with lysosomes, degrading its content (Futter et al., 1996). The degradative pathway is not only used by signaling receptors to terminate signaling but also for the degradation of lipids. For example, Rab8 is involved in the regulation of cholesterol efflux via this pathway, an important step for myelination (Linder et al., 2009; Zhou et al., 2019).

Interestingly, the ErbB pathway, a crucial pathway for Schwann cell myelination, relies heavily on proper degradation and recycling. Neuregulin1 signaling through ErbB leads to Schwann cell myelination, both ErbB2 and ErbB3 are expressed in Schwann cells (Michailov et al., 2004; Taveggia et al., 2005). However, both receptors follow different pathways in this process- ErbB2 is recycled, whereas ErbB3 is degraded via the ESCRT pathway. Ubiquitinated ErbB recognizes ESCRT-0, recruits ESCRTI-III and is subsequently incorporated into the invaginations—which bud off ILVs within MVBs (for a good review please see Lee et al., 2017). This process attenuates signal transduction and ends in receptor degradation. Abnormalities in this pathway again lead to increased neurotoxic waste and apoptosis.

Abnormalities in lysosomal degradation have been reported for mutations in *PMP22, LITAF, RAB7, DYNC1H1, LRSAM1, MTMR2,13,5, NDRG1*, and *FIG4* causing CMT1A, 1C, 2B, 2O, 2P, 4B, 4D, and 4J, respectively.

## Autophagy

Another degradative pathway is the one of autophagy. A pathway mostly used for the degradation of aggregated and misfolded proteins but also whole organelles (like mitochondria, termed mitophagy), induced by cellular stress. For this, a double membrane structure called phagophore forms, expands and closes upon itself to generate an autophagosome engulfing protein aggregate. The autophagosome then fuses with a lysosome leading to the degradation of its content and a subsequent nutrient release into the cytosol (also referred to as macroautophagy). Rab1, 4, and 11 are involved in the membrane delivery for the formation of the phagophore, whereas autophagy receptor p62 binds ubiquitinated cargo and Microtubule-associated protein 1A/1B-light chain 3 (LC3) on autophagosomes (Komatsu et al., 2007; Kiral et al., 2018). The class 3 phosphoinositide 3-kinase (PI3K) complex, recruited by Rab5, produces PI_3_P for the nucleation of the phagophore, whereas Rab33 is involved in the elongation. Autophagosomes fuse with late endosomes to so called amphisomes that traffic retrogradely- possibly by the same Rab7/Rab-interacting lysosomal protein (RILP)/dynactin complex that regulates the transport of late endosomes (Jordens et al., 2001; Cheng et al., 2015). Rab7 and Rab24 then mediate the fusion with lysosomes (Kiral et al., 2018). The autophagic flux depends on the autophagosomal transport, and fusion with lysosomes. In healthy neurons, few autophagosomes are observed indicating a low level of autophagy or a very quick turnover (Mizushima et al., 2004; Boland et al., 2008).

Autophagy related proteins have also been shown in Schwann cells, where autophagy plays an important role for cell plasticity, myelin maturation, and compaction. Excess cytoplasm is removed by autophagy leading to more compact myelin. Furthermore, Schwann cells rely on autophagy after nerve injury for the phagocytosis of myelin debris (Belgrad et al., 2020).

Alterations in autophagy were seen with mutations in *PMP22, RAB7, HSPB*1, and *FIG4* causing CMT1A, 2B, 2F, and 4J, respectively.

## Mitochondria Transport

Neuronal cells have a relatively large energy consumption, which is why they rely heavily on a functioning mitochondria system. Trafficking is an important process for mitochondrial maintenance. Mitochondria need to be transported to regions of high energy demand. If mitochondria transport is disturbed this would lead to a local energy crisis and to degradation of the long neurons of the PNS. Transport is regulated by a Miro/Milton complex (Stowers et al., 2002; Fransson et al., 2006; Glater et al., 2006). Miro binds the outer mitochondrial membrane via its carboxyterminal, whereas Milton is the adaptor protein of Miro and the motor proteins. Accumulated damaged mitochondria are degraded by mitophagy as described above. Disruptions in mitochondria transport and location have been described in many sub-types of CMT including mutations in *MFN2* (CMT2A2), *RAB7* (CMT2B), *GARS1* (CMT2D), *NEFL* (CMT2E), *HSPB1* (CMT2F), and *DYNC1H1* (CMT2O). For a recent review on mitochondria transport in a disease context refer to (Schiavon et al., 2021).

## Cytoskeletal Basis for Transport

The basic structure or architecture of neurons is provided by its cytoskeleton, which also serves as the tracks for intracellular trafficking. The cytoskeleton is comprised of three building blocks, microfilaments, intermediate filaments, and microtubules. Microfilaments are made by actin and are mostly found in mobile/changing structures like the growth cone or newly formed synapses. Intermediate filaments are made from three different neurofilaments (light, medium, and heavy polypeptide) and peripherin in the PNS. The filaments share a structural organization of a central α-helical rod that drives assembly of dimers, protofilaments, and filaments of 10 nm diameter (Laser-Azogui et al., 2015). Neurofilaments are the most abundant cytoskeletal component in myelinated axons. They have the intrinsic role to form and maintain the axonal architecture, its diameter and the intracellular transport of cargo in dendrites. Posttranslational modifications influence neurofilament assembly, hence the axonal size of large myelinated axons.

Lastly, microtubules are composed of α and β tubulin forming profilaments, where 13 profilaments make 1 microtubule with a diameter of about 25 nm (Prior et al., 2017). Microtubules, also referred to as molecular tracks, are modified by acetylation and detyrosination, which influences trafficking. Acetylation increases the binding of motor proteins thus enhancing transport and is highest in stable and long-lived microtubules, like axons (Reed et al., 2006). Acetylation of α-tubulin improves the binding capacities of kinesins to the microtubules thus enhancing transport (Reed et al., 2006) but has also shown to increase dynein recruitment to microtubules (Dompierre et al., 2007). Whereas, detyrosination guides kinesin-1 to the axon (Konishi and Setou, 2009) and regulates dynein based transport (Nirschl et al., 2016).

Kinesins are the motor proteins involved in the plus-end directed (anterograde) transport. Dyneins are involved in minus-end directed or retrograde transport. Dynein interacts with cargo via a dynein/dynactin complex involving p150glued. Deregulation in microtubule assembly and disassembly has been shown to affect axonal growth (Markworth et al., 2019). Importantly, neurotrophic signaling can affect microtubule stability (Goold and Gordon-Weeks, 2003).

Many types of CMT have shown to have cytoskeletal abnormalities (Brownlees et al., 2002), including mutations in *PMP22* (CMT1A), *MFN2* (CMT2A2), *RAB7* (CMT2B), *GARS1* (CMT2D), *NEFL* (CMT2E), *HSPB1* (CMT2F), *FIG4* (CMT4J), *FGD4* (CMT4H), *DNM2* (DI-CMT), and *GJB1* (CMT1X). Whether these are causative or secondary remains to be shown in many cases.

## Phospholipids

Finally yet importantly, while the phospholipid system is involved in every step of intracellular trafficking, it is worthy of its own section. As already mentioned, the endosomal trafficking journey begins with the synthesis of PI_4_,_5_P_2_ at the site of endocytosis and changes over the course of endosomal maturation, making phospholipids not only a marker for endosomal maturation but an important regulator of the endosomal system (Wallroth and Haucke, 2018). Early endosomes are mostly comprised of PI_3_P, generated by class III phosphoinositide VPS34, which, in turn, is recruited by Rab5. PI_3_P is then recognized by the FYVE domain of ESCRT-0 subunit Hrs, which is recruited to the endosomes to promote ESCRT sorting and MVB formation. During endosomal maturation PI_3_P is phosphorylated by PIKFYVE complex/PI5K to PI_3_,_5_P_2_. PI regulating enzymes can affect endosome to lysosome trafficking and therefore inhibit the degradation of cell surface proteins (Berger et al., 2011).

Interestingly, as a component of the plasma membrane, phospholipids have been shown to play an important role in Schwann cells too. More precisely, phospholipids on Schwann cells have shown to be regulators of compact myelination. As PI3K generates PI_3_,_4_,_5_P_3_ at the plasma membrane, which then activates the Akt pathway, a promyelinating signaling cascade in the PNS, that is tightly regulated by phosphatase and tensin homolog (PTEN), downregulating PI_3_,_4_,_5_P_3_ (Lee et al., 2017). This again shows that phosphoinositides do not only function as membrane markers for different organelles, but also hold a signaling and regulating function in the PNS. This makes them interesting candidates to investigate in axonal and demyelinating CMT as a deregulation would affect the whole intracellular trafficking pathway spanning from endocytosis to degradation.

Specifically mutations in *MTMR2,13,5* and *FIG4* causing CMT4B and CMT4J, respectively, have shown to disrupt the phospholipid system.

## CMT Subtypes Showing Impaired Intracellular Trafficking

While genetic mutations leading to CMT also affect other cellular processes (e.g., genes coding for human aminoacyl tRNA synthetases such as *GARS1* [glycyl-tRNA synthetase (GlyRS)], which catalyzes the first step of protein synthesis), eventually many processes meet at the platform of intracellular trafficking. For example, CMT2D-associated mutants of GlyRS stimulate deacetylase activity on α-tubulin (Mo et al., 2018). Further, even protein synthesis is linked to intracellular trafficking: mRNAs made in the nucleus associate with so-called RNA-binding proteins (RBPs). These RBPs are transported as ribonucleoprotein particles (RNPs) to distal subcellular locations for local translation. To perform local translation, RNPs associate with late endosomes/lysosomes, which are transported along the axons. Finally, These RNP-associated late endosomes have been found to dock at mitochondria sustaining mitochondrial health (Cioni et al., 2019). Intriguingly, this process is disrupted in CMT2B, caused by mutations in the late-endosomal Rab7-GTPase (Cioni et al., 2019). Therefore, rather than listing CMT-causing mutations based on their functions, we deciphered where and how, to today’s knowledge, CMT mutations affect intracellular trafficking and how this then could explain the observed phenotypes.

The length, polarity and post-mitotic state make peripheral sensory and motor neurons extremely dependent on intracellular axonal transport for proper function and maintenance. It is therefore understandable that even mutations in ubiquitously expressed proteins cause a cell type specific phenotype in these neurons. Importantly, also subtypes that show both, axonal and demyelinating phenotypes show many mutations involved in intracellular transport in neurons but also in Schwann cells, hindering proper myelination.

Schwann cells have a highly polar structure and rely heavily on membrane transport for myelination. This can be seen in the involvement of the intracellular transport machinery in the pathology of demyelinating CMT1 as described below. Although many other factors are involved in the pathology, the involvement of intracellular traffic in CMT is intriguing as it highlights the importance of the intracellular transport machinery for many aspects of proper cellular function. Further, it sheds light on the importance of considering intracellular dynamics when looking for potential disease mechanisms. The fluidity yet tight regulation of this system is its toughest aspect to study. However, the following examples highlight the importance of considering this system as highly dynamic when interpreting static results. The CMT subtypes discussed in this review and their corresponding trafficking phenotypes are summarized in Table 1.

**TABLE 1:** An overview of the CMT mutations involved in intracellular trafficking discussed in this review, including their relevant phenotypes.

Type	*Gene symbol*	Gene name	Trafficking phenotype	References
CMT1A	*PMP22*	Peripheral myelin protein 22	Autophagy/saturation of proteasome, disrupted PMP22 transport, elevated ErbB levels, reduction in slow axonal transport, altered cytoskeletal organization and NF phosphorylation	De Waegh and Brady, 1990; de Waegh et al., 1992; Massa et al., 2006; Fortun et al., 2007; Rangaraju et al., 2010; Marinko et al., 2020
CMT1C	*LITAF*	Lipopolysaccharide induced TNF factor	Mislocalization from early endosomes to cytosol, reduced ESCRT recruitment, decreased EGFR degradation, enlarged endosomes/lysosomes	Lee et al., 2011, 2012; Edgar et al., 2020
CMTX	*GJB1*	Gap junction protein beta 1	Dysfunctional anterograde trafficking of GJB1, defects in retrograde axonal transport and neurofilament phosphorylation	Deschênes et al., 1997; Yum et al., 2002; Abrams et al., 2003; Kleopa et al., 2006; Vavlitou et al., 2010
CMT2A1	*KIF1B*	Kinesin family member 1B	Defects in IGFR1 transport, perinuclear accumulation and defects in anterograde cargo transport	Zhao et al., 2001; Xu et al., 2018
CMT2A2	*MFN2*	Mitofusin 2	Disturbed mitochondrial transport, mitochondria aggregation in proximal segment, loss of tubulin acetylation	Baloh et al., 2007; Saporta et al., 2015; Bernard-Marissal et al., 2019; Picci et al., 2020
CMT2B	*RAB7A*	Rab7A, Member RAS oncogene family	Altered retrograde traffic, altered TrkA/EGFR signaling, decreased RILP levels, increased peripherin interaction, disturbed retromer binding, reduced autophagic flux	Spinosa et al., 2008; Seaman et al., 2009; Basuray et al., 2010; BasuRay et al., 2013; Cogli et al., 2013; Zhang et al., 2013; Janssens et al., 2014; Colecchia et al., 2018
CMT2D	*GARS1*	Glycyl-tRNA synthetase 1	Reduced acetylated tubulin levels, disrupted mitochondria transport, novel binding to HDAC6 and TrkA, disrupted NGF transport, increased interaction with neuropilin-1→ disrupted interaction with VEGFR	He et al., 2015; Kim et al., 2016; Sleigh et al., 2017; Benoy et al., 2018; Mo et al., 2018
CMT2E/CMT1F	*NEFL*	Neurofilament light	Disrupted anterograde traffic of NF-L (aggregation/accumulation) and mitochondria	Brownlees et al., 2002; Pérez-Ollé et al., 2005; Saporta et al., 2015
CMT2F	*HSPB1*	Heat shock protein family B (small) member 1	Intracellular aggregates NF-M, disturbed retrograde mitochondrial transport, increased binding to α-tubulin, reduced autophagic flux	Ackerley et al., 2006; Almeida-Souza et al., 2011; D’Ydewalle et al., 2011; Kim et al., 2016; Kalmar et al., 2017; Haidar et al., 2019
CMT2O	*DYNC1H1*	Dynein cytoplasmic 1, heavy chain 1	Decreased retrograde transport of lysosomes and trophic factors, reduced mitochondrial transport	Hafezparast et al., 2003; Perlson et al., 2009; Ori-McKenney et al., 2010; Zhao et al., 2016
CMT2P/CMT2G	*LRSAM1*	Leucine-rich repeat- and sterile alpha motif-containing 1	Altered interaction with ESCRT protein TSG101 and altered EGFR degradation	Guernsey et al., 2010; Palaima et al., 2021
DI-CMTB	*DNM2*	Dynamin 2	Blocks dynamin-dependent endocytosis, defects in microtubule stability	Tanabe and Takei, 2009; Sidiropoulos et al., 2012
CMT4B1	*MTMR2*	Myotubularin related protein 2	GluR2 uptake increases upon loss of functional protein, also lack of recycling initiation/missorting into lysosomes, altered EGFR degradation	Bolino et al., 2004; Cao et al., 2008; Lee et al., 2010; Berger et al., 2011
CMT4B2	*MTMR13*	Myotubularin related protein 13		
CMT4B3	*MTMR5*	Myotubularin related protein 5		
CMT4C	*SH3TC2*	SH3 domain and teratricopeptide repeats 2	Decreased ErbB2 internalization, decreased interaction with Rab11, increased recycling of TF	Arnaud et al., 2009; Roberts et al., 2010; Gouttenoire et al., 2013
CMT4D	*NDRG1*	N-myc downstream regulated 1	Disturbed Rab4 endosomes, disturbed TFR, e-cadherin, ErbB and LDL recycling, abnormal endosomal maturation, delayed fusion of MVBs with lysosomes	Kachhap et al., 2007; Pietiäinen et al., 2013; Li et al., 2017
CMT4H	*FGD4*	FYVE, RhoGEF and PH domain containing 4	Defective TF internalization, alterations in microfilament structure	Delague et al., 2007; Horn et al., 2012
CMT4J	*FIG4*	Fig4 phosphoinositide 5-phosphatase	Decreased levels of PI3,5P2 on endosomes, enlarged endosomes and lysosomes, decreased autophagic flux, defective cholesterol transport	Ferguson et al., 2009; Vaccari et al., 2015; Bharadwaj et al., 2016

## Axonal CMT2

### CMT2A1-KIF1Bβ

In CMT2, several defects in intracellular trafficking have been reported (Figure 1).

CMT2A1 is caused by mutant KIF1Bβ, a kinesin family member that plays a role in neuronal survival and function due to its role in anterograde transport (Zhao et al., 2001). KIF1Bβ directly binds to insulin like growth factor (IGF) receptor (IGFR) 1β, a receptor tyrosine kinase that signals for axonal outgrowth via IGF-I signaling and activation of the PI3K-Akt pathway (Laurino et al., 2005; Scolnick et al., 2008). In one of the studied CMT2A1 mutations (Y1087C) binding of KIF1Bβ to IGFR is significantly reduced, leading to less IGF1R transport, reduced surface expression and reduced axonal outgrowth in mouse hippocampal neurons (Xu et al., 2018). Further, KIF1Bβ is involved in the development of myelinated axons, both in the CNS and PNS (Lyons et al., 2009 in Xu). Other mutations have been reported in the conserved ATP binding site of the kinesin motor (Q98L), leading to a perinuclear accumulation of the protein and defects in cargo transport. This defect in transport was visible by decreased levels of synaptic vesicle proteins including synaptotagmin and synaptic vesicle protein 2 (SV2) in western blots of proximal and distal nerve sections after sciatic nerve ligation of heterozygous *kif1B*± mice in comparison to *kif1B*+/+ mice (Zhao et al., 2001). This study concluded a trafficking defect due to decreased protein levels on western blots of lysed nerve sections comparing distal and proximal parts. Levels of synaptotagmin and SV2 were reduced in both, distal and proximal section of the heterozygous mice compared to control. While the authors concluded a Kif1B- dependent trafficking defect, decreased levels could also occur from downregulation of these proteins or altered degradation instead of altered trafficking. Therefore, to specifically pinpoint trafficking defects, live-imaging using trafficking markers would help to decipher this question. In addition, KIF1Bβ is able to bind to other cargo. Therefore, it would be interesting to see if other intracellular transport systems are also affected, including the transport and signaling of epidermal growth factor receptor (EGFR), mitochondrial transport and autophagic turnover as presented for other CMT types (see below). Lastly, the effect on IGF1R trafficking and signaling in other CMT types would be intriguing to unwrap, to see if it is a common denominator or if commonalities can be found in the effect on the downstream signaling cascade.

### CMT2A2

The most common type of CMT2 is CMT2A2, caused by dominantly inherited point mutations in *Mitofusin 2* (*MFN2*; Züchner et al., 2004). MFN2 is a dynamin family GTPase located on the outer mitochondrial membrane protein and part of the Miro/Milton tethering complex, tethering mitochondria to kinesin. MFN2 is further involved in mitochondria morphology, fusion and motility, endoplasmic reticulum tethering and synapse formation (Chen et al., 2003; De Brito and Scorrano, 2008; Misko et al., 2010). CMT2A2 mutations cause a decrease between mitochondria and endoplasmic reticulum tethering and a reduction in neurite length of primary motor neurons. Live cell imaging also revealed fewer mitochondria in the sciatic nerve axons with a higher proportion of very slow-moving mitochondria compared to control (Bernard-Marissal et al., 2019). This is in line with data from primary sensory neurons, where overexpressed mutant MFN2 led to a loss of mitochondria in distal and an aggregation in the proximal axonal segments with fewer mobile mitochondria (Baloh et al., 2007). The phenotype of slower mitochondrial movement was also observed in spinal motor neurons derived from patient induced pluripotent stem cells, but to lesser extent (Saporta et al., 2015). In a CMT2A2 mouse model, loss of mitochondria was observed in the distal part of sciatic nerve axons as well as a loss of tubulin acetylation that worsened with age (Picci et al., 2020). However, since these mice display a neuropathic phenotype before the decrease of acetylation is detected, another pathomechanism has to be in play. Yet treatment with an histone deacetylase 6 (HDAC6) inhibitor ameliorated some of the motor and sensory phenotypes in these mouse models (Picci et al., 2020). It would be very interesting to see if and how the treatment affected mitochondrial transport and how it acted on a molecular level by applying it *in vitro.* This, in turn, would help to put this finding in context with previous studies focusing on the molecular pathomechanisms [like they did for CMT2D causing GlyRS (Benoy et al., 2018)]. Still, the questions that remain are: is the deacetylation of tubulin is a cause of the decreased mitochondrial transport? Would the deacetylation then, in turn, amplify the trafficking defect by decreased motor protein association, or do the pathomechanisms work in parallel? If mitochondria can no longer be transported to sites of high energy demand, like synapses or nodes of Ranvier, this would lead to a local energy crisis and explain neuronal malfunction in the longest and most distal parts as seen in CMT2A. Therefore, future studies on mitochondrial transport, or how to overcome disruptions in kinesin tethering would be an interesting point of investigation.

### CMT2B

Autosomal dominant CMT2B, marked by primarily axonal degeneration, is caused by mutations in the late Rab7 GTPase. As of today 6 mutations are known to cause CMT2B, with all mutations being located close to the GTP binding pocket altering the binding kinetics (Spinosa et al., 2008; McCray et al., 2010; Saveri et al., 2020). Rab7 has been shown to transport ligand bound neurotrophic receptors TrkA and TrkB retrogradely to the soma (Deinhardt et al., 2006). Even though the CMT2B-causing Rab7 mutations are no loss of function mutations, alterations in retrograde traffic were observed in a drosophila model and in cultured mouse dorsal root ganglia overexpressing the mutant Rab7 proteins (Zhang et al., 2013; Janssens et al., 2014). The binding of Rab7 and TrkA was not changed by the mutations. However, signaling of TrkA and EGFR was altered in cells expressing CMT2B mutant Rab7, possibly as a result of defective signaling from endosomes (Maxfield and Yamashiro, 1987; Rink et al., 2005; Marat and Haucke, 2016; Bakker et al., 2017)—since signaling from the plasma membrane was still intact (Basuray et al., 2010; BasuRay et al., 2013). In addition, the different mutants showed decreased expression of RILP, a Rab7 effector important for lysosomal transport by recruiting dynein-dynactin motors, as well as an increased interaction with peripherin, an intermediate filament of the PNS (Spinosa et al., 2008; Cogli et al., 2013). Further, one of the mutants showed disturbed binding to the protein sorting complex retromer by co-immunoprecipitation, suggesting a pathomechanism that involves mis-sorting of receptors on their trafficking route (Seaman et al., 2009). A study from 2018 also reported alterations in autophagy in CMT2B. All tested mutants displayed reduced localization on autophagic compartments and reduced autophagic flux in HeLa cells, similarly to a dominant negative mutant of Rab7. Further, basal and induced autophagy were decreased in skin fibroblasts from a CMT2B patient (Colecchia et al., 2018; Romano et al., 2020). As mentioned, local translation by RNPs localizing to late-Rab7 endosomes and mitochondria is disrupted in CMT2B (Cioni et al., 2019). The diverse phenotypes of CMT2B- causing mutations in Rab7 is explainable by the involvement of Rab7 in many aspects of this highly dynamic system (e.g., transport, signaling of receptors, degradation, local translation), which makes pinpointing the causal effect of mutant Rab7 extremely difficult. It would therefore be very interesting to study if within these mechanisms some common modalities are present—e.g., do CMT2B-causing mutations have difficulties binding to dynein which would disrupt trafficking, signaling, maturation and degradation.

### CMT2D

Mutant GlyRS encoded by *GARS1* causes autosomal dominant CMT2D and distal hereditary motor neuronopathy (Antonellis et al., 2003). A mutant mouse model shows reduced acetylated α-tubulin levels and disrupted mitochondrial transport (Benoy et al., 2018; Mo et al., 2018). How the molecular mechanism of tRNA generation influences intracellular trafficking is not yet understood. To date, GlyRS mutants have been found to show novel and increased binding properties to cytoskeletal proteins and receptors (He et al., 2015). Mutant but not wild type GlyRS co-immunoprecipitates with HDAC6. HDAC6 deacetylases tubulin leading to unstable tubulin. Therefore, binding of mutant GlyRS to HDAC6 is believed to increase the deacetylase activity. A HDAC6 specific inhibitor has been found to improve mitochondrial axonal transport and relieve both, motor and sensory systems in mice (Benoy et al., 2018; Mo et al., 2018). The similarity to MFN2 causing CMT2A2 is striking, yet needs to be confirmed by producing more comparable studies and results. Both mutant MFN2 and GlyRS show reduced acetylated tubulin levels and decreased mitochondrial transport (Baloh et al., 2007; Benoy et al., 2018; Mo et al., 2018; Picci et al., 2020). Further, mouse models of each show an improvement of motor and sensory phenotypes upon treatment with HDAC6 inhibitors. How mutations in MFN2 lead to decreased acetylated tubulin, which in turn could lead to decreased binding of motor proteins to it and therefore to decreased transport of cargo/mitochondria, is still open. It is possible that MFN2 has a direct impact on the acetylation levels or that it is a secondary effect to the disrupted mitochondrial transport. It would therefore, be of interest if mitochondrial transport was improved upon HDAC6 inhibitor treatment in models of CMT2A2 (MFN2) or if deliberately disrupting mitochondrial transport leads to a decrease in tubulin acetylation.

GlyRS also shows increased interactions with receptors. GlyRS binds neuropilin-1, a co-receptor of the vascular endothelial growth factor receptor (VEGFR). This interaction is enhanced by the GlyRS-CMT2D causing mutations. The increased binding of mutant GlyRS to neuropilin-1 disrupts its interaction with VEGFR. While genetic depletion of neuropilin-1 worsens the CMT phenotype, overexpression of VEGF improves motor problems (He et al., 2015), indicating that this pathway is also involved in the pathogenesis. Interestingly, VEGF signaling has been shown to be regulated by intracellular trafficking (Horowitz and Seerapu, 2012). Whether the altered VEGF pathway contributes to the pathology in parallel or in stream with the altered tubulin acetylation remains unknown. To pinpoint the effects of VEGFR signaling in CMT2D, trafficking and downstream signaling could be followed. For example, does VEGFR change its downstream targets since neuropilin-1 has been found to act as a “gating” protein switching downstream signaling of PlexinD1 to VEGFR2 (Chauvet et al., 2007)?

Lastly, mutant GlyRS but not wild type GlyRS binds to TrkA. There is a correlation between binding intensity and disease severity. Even though the molecular effect on Trk binding is not clear, an increased Trk activation by increased Erk1/2 phosphorylation is indicated (Sleigh et al., 2017). Whether the disrupted transport of NGF (Mo et al., 2018) is a consequence of altered tubulin acetylation and transport defects in general or due to GlyRS’ increased binding to NGF-receptor TrkA remains to be answered. The causal relationship of growth factor trafficking and tubulin acetylation needs to be determined. It is known that neurotrophic factor signaling affect microtubule dynamics (Goold and Gordon-Weeks, 2003). Other options are changes in downstream signaling due to increased TrkA/GlyRS binding.

### CMT2E

Mutations in *NEFL* are quite intriguing as they cause mostly axonal CMT2E but can also be classified as CMT1F because severely reduced nerve conduction velocity is a reported phenotype. However, the decreased conduction velocity that usually stems from demyelination is proposed to be caused by a decrease in axonal diameters in CMT2E, making it ultimately axonal (Lancaster et al., 2018). The mutations associated with CMT2E/1F are most commonly missense mutations in the head domain or in the central rod domain of neurofilament light polypeptide (NEFL; Mersiyanova et al., 2000; De Jonghe et al., 2001; Lerat et al., 2019). Mutations disturb axonal transport of NEFL that is generated in the soma and transported along the axon, shown by lack of NEFL in the distal part of axons and accumulation in the proximal part and soma (Brownlees et al., 2002). However, live cell imaging of fluorescently tagged mutant NEFL in primary rat neurons did not show any alteration of NEFL movement compared to WT indicating that NEFL traffic greatly depends on the model being used (Stone et al., 2019). In contrast, transport of mitochondria is disrupted—seen in an accumulation of mitochondria in the cell body region and loss in the distal axon of sympathetic neurons (Brownlees et al., 2002; Pérez-Ollé et al., 2005). In patient derived iPSCs differentiated into spinal motor neurons, mitochondria movement was reported to be slower and shorter distanced (Saporta et al., 2015). Aggregates of mutated NEFL were observed in transfected rat cortical neurons and mouse models, as seen for other neuropathies. However, patient nerve biopsies and patient derived motor neurons only showed disorganized NEFL polymer accumulation not aggregation (Brownlees et al., 2002; Fabrizi et al., 2007; Adebola et al., 2015; Saporta et al., 2015; Zhao et al., 2017). Overall, above studies should be carefully interpreted since data arose from different model systems—as phenotype depends greatly on the accompanying neurofilament proteins available for co-assembly (Stone et al., 2019).

### CMT2F

Similar effects as described for CMT2D (mutant GlyRS) on α-tubulin acetylation and axonal transport have also been reported for CMT2F, which is caused by a mutation in heat shock protein family B (small) member 1 (*HSPB1)*. CMT2, causing HSPB1 has been found to cause intracellular aggregates of components including neurofilament medium polypeptide (NEFM) and p150 dynactin (Ackerley et al., 2006). In sensory and motor neurons, expression of mutant HSPB1 lead to disturbed retrograde mitochondrial transport, whereas axonal transport of neurotrophic factor p75 was only minimally affected, indicating a cargo specific defect (D’Ydewalle et al., 2011; Kim et al., 2016; Kalmar et al., 2017). This mitochondrial transport defect was partially rescued by an HDAC6 inhibitor. Almeida-Souza et al. (2011) have shown that mutant HSPB1 reveals an enhanced binding efficiency to α-tubulin leading to stabilization of microtubules without changing the acetylation pattern of tubulin. This seems counterintuitive to the beneficial effect of HDAC6 inhibitors and shows that more studies are needed to figure out the molecular pathway of HSPB1 and HDAC6 inhibitors in the cytoskeleton. Interestingly, the commonalities of phenotypes between CMT2D (*GARS1*), CMT2E (*NEFL*), and CMT2F (*HSPB1*) all share a common defect: disruption in α-tubulin acetylation. A comparison on tubulin dynamics and structure could reveal additional shared phenotypes and increase the understanding of how these common phenotypes occur despite different genes affected. Furthermore, starvation induced autophagic flux was reduced in patient derived motor neurons. The exact molecular pathway of how HSPB1 is involved in autophagy remains to be answered but it has been shown that HSPB1 binds to the autophagy inducing receptor SQSTM1 and that this binding is increased by some mutants of HSPB1. Those mutants failed to induce autophagic pores upon starvation (Haidar et al., 2019).

### CMT2O

CMT2O is an autosomal dominant type of CMT, caused by a His306Arg mutation of dynein cytoplasmic 1 heavy chain 1 (*DYNC1H1*). The His306Arg mutation resides in the highly conserved residue of the homodimerization domain of DYNC1H1 (Weedon et al., 2011). As dynein is the main motor protein responsible for minus-end/retrograde transport, a pathomechanism involving defective axonal transport is expected. Indeed, tagged dynein from the Loa mice line, which carries a mutation in the binding domain of DYNC1H1, show decreased run length of retrograde transport of lysosomes (Hafezparast et al., 2003; Ori-McKenney et al., 2010) as well as decreased transport of trophic factors in a sciatic nerve ligation assay (Perlson et al., 2009). In mouse models with a 9bp deletion mutation in the stem domain of DYNC1H1 (responsible for cargo binding and homodimerization), retrograde axonal transport of NGF was reduced, which caused increased apoptosis upon NGF stimulation at the peripheral axon (Zhao et al., 2016). Whether the downstream signaling cascade of TrkA, the receptor for NGF, was altered as reported for mutated Rab7 and GlyRS has not been investigated yet. Further, mitochondrial transport is reduced in these mice as also reported for mutated MFN2, GlyRS and NEFL. The model shows that DYNC1H1 has a crucial role in the transport of both neurotrophic factors and mitochondria a defect shared among several CMT subtypes.

### CMT2P

Recessive and dominant axonal CMT2P are caused by mutations in *LRSAM1* (leucine rich and sterile alpha motif containing) encoding a universally expressed RING-type E3 ubiquitin ligase (Guernsey et al., 2010). Thus far, its only known target is TSG101, a member of ESCRT I, involved in the degradation pathway of EGFR (Palaima et al., 2021). Though only known since the last decade, first results already show the involvement of LRSAM1 in the intracellular trafficking pathway and more results regarding its exact involvement in the molecular processes of EGFR degradation and possible other trafficking phenotypes are to be expected in the future.

In summary, the most prominent shared defects in axonal CMT include abnormalities in mitochondrial transport, transport of neurotrophic factors, tubulin acetylation and altered autophagy. These mechanisms, in turn, could affect downstream signaling from endosomes as well as microtubule dynamics via trophic signaling (Goold and Gordon-Weeks, 2003), One downside of interpretation is that experiments conducted vary between model systems used. Further, the level of detail in how the transport mechanisms are studied also shows variability. Therefore, comparative studies focusing on the effect of the different CMT mutated proteins would be ideal to identify common pathologies. Further, checking for phenotypes reported in one subtype (like altered IGFR transported as reported for CMT2A1) in other subtypes will complete the picture of possible shared mechanisms. The same holds true for CMT subtypes not mentioned in this review because not enough data to altered trafficking mechanism were reported as of yet.

## CMT Subtypes With Demyelinating and Axonal Phenotypes

Importantly, not only predominantly axonal types of CMT show intracellular transport defects. In fact, several of the demyelinating subtypes have shown abnormal trafficking including CMT1, the most common type of CMT, as well as CMT1X, CMT4, and DI-CMT (Figures 1, 2). This indicates that trafficking is also a crucial step in Schwann cells. Of note is that the majority of trafficking defects in CMT subtypes with more demyelinating phenotypes is seen in the endocytic and recycling pathway indicating these two steps critical in Schwann cell myelination. DI-CMT, CMT4B, 4C, 4D, and 4H all show endocytic alterations, recycling defects or both. On the other hand, CMT4B and CMT4J affect phosphoinositide compositions altering endocytic processes, recycling, maturation, and degradation pathways. This shows that the PI regulation is essential for the complete dynamic process of intracellular trafficking and cannot be pinpointed to a single subsection.

### DI-CMTB

Mutations in dynamin 2 cause dominant-intermediate CMT (DI-CMTB) displaying both axonal and demyelinating phenotypes, sometimes also classified as axonal CMT2M (Züchner et al., 2005). Dynamin 2 is a ubiquitously expressed fission protein, responsible for the fission of intracellular vesicles after endocytosis and for the fission from endosomes. The mutations causing Di-CMTB are located in the Pleckstrin homology domain of dynamin 2, which binds to PI_4_,_5_P_2_ required for membrane localization. One of the DI-CMTB causing mutants (K558E) blocks dynamin-dependent endocytosis in a dominant negative fashion, whereas another (551Δ3) showed defects in microtubule stability, indicating two different pathogenic mechanisms (Tanabe and Takei, 2009). Of note, dynamin 2 also plays a role in receptor trafficking as it is involved in targeting receptors into the recycling pathway from early endosomes (Jovic et al., 2010). Therefore, defects in dynamin 2 could affect receptor signaling by blocking receptor endocytosis as well as re-activation if receptor re-insertion to the plasma membrane is disrupted. Although live cell imaging experiments are sparse for DI-CMTB, the disturbances in three of the main fundaments for intracellular transport—endocytosis, recycling, and microtubules, could lead to trafficking defects and would be ideal candidates to receive further investigation. Interestingly, dynamin 2 is essential for Schwann cell myelination of the peripheral nerves. An induced dynamin 2 deletion in adult Schwann cells leads to a demyelinating neuropathy (Gerber et al., 2019). However, it is unclear if this defect is caused by disrupted endocytosis as seen by a Transferrin uptake assay, or caused by altered levels of ErbB2 receptors on the plasma membrane (Sidiropoulos et al., 2012). The involvement of dynamin 2 in Schwann cell myelin maintenance provides a basis for the intermediate pathogenesis seen in DI-CMTB.

### CMT4B

CMT4 is a rather rare subtype of the disease mostly inherited in an autosomal recessive pattern characterized by myelin deformities and a relatively early onset.

CMT4B is caused by autosomal recessive mutations in three of the myotubularin-related protein (MTMR) family of phosphoinositide 3-phosphatases with a mostly demyelinating phenotype with focal hypermyelination. CMT4B1 is caused by loss-of-function mutations in the catalytically active MTMR2, whereas CMT4B2 and CMT4B3 are caused by mutations in catalytically inactive MTMR13 and MTMR5, respectively (Bolino et al., 2000; Berger et al., 2002; Robinson and Dixon, 2005). MTMR2 dephosphorylates PI_3_P (mainly present on early endosomes) to PI, and PI_3_,_5_P_2_ (mainly present on late endosomes) to PI_5_P. MTMR5 and MTMR13 directly interact with active MTMR2 and increase its catalytic activity as well as recruit MTMR2 to membrane compartments (Kim et al., 2003; Robinson and Dixon, 2005). Due to faulty phosphatase activity, PI_3_P and PI_3_,_5_P_2_ are predicted to accumulate on endosomes in CMT4B.

As discussed above, phosphoinositides are an important regulator of many steps along the intracellular trafficking pathway, as well as of myelination. Therefore, it is of no surprise that several studies have found effects of CMT4B mutations in many different aspects of the intracellular pathways. Even though expression of MTMR in Schwann cells is very low (Berger et al., 2002), mutations in Schwann cells is sufficient to induce CMT4B-like pathology (Bolis et al., 2005).

A study in cortical neurons has located MTMR2 to synapses by interaction with PSD95. Here, MTMR2 seems to function as a negative regulator of endocytosis, as the uptake of AMPAR subunit GluR2 increases upon loss of functional protein (Lee et al., 2010). As MTMR2 has been shown to interact with SAP97/Dlg1 a part of the PSD family in Schwann cells, it is plausible for MTMR to play a regulatory role of endocytosis in Schwann cells (Bolino et al., 2004). Whether this process contributes to the pathology remains to be answered.

Further, loss of MTMR2 promotes the sorting of internalized AMPA receptors to lysosomes, indicating that active MTMR2 plays a role in preventing AMPA degradation, possibly by initiating a recycling pathway (Lee et al., 2010). However, in epithelial cells, knockdown or overexpression of MTMR2 leads to a blockage of EGFR degradation *in vitro* in two different studies, implicating MTMR directly in the degradative pathway (Cao et al., 2008; Berger et al., 2011). Further, MTMR2 shows binding to PI3K adaptor hVPS34/hVPS15 complex that also interacts with Rab7, indicating a possible link in pathology of CMT4B and CMT2B. Although the direct effect of CMT4B mutations on the degradative pathway are not shown, the general involvement of MTMR in the degradative pathway offers many possible pathomechanisms. Normally, MTMR2 recruits RME8 via PI_3_P, which, in turn, regulates EGFR traffic from endosomes to lysosomes—a pathway that could be disrupted in CMT4B (Xhabija et al., 2011). Overall, CMT4B shows that misregulation of phospholipid composition can lead to disruptions in endocytosis, recycling and degradation. Since CMT4B shows predominantly demyelinating phenotypes, it will now be interesting to see how CMT4B mutations affect ErbB endocytosis, recycling and degradation in Schwann cells, considering that this is a major signaling pathway for myelination, as well as its downstream promyelinating PI3K/Akt signaling cascade. Interestingly, Akt levels were altered in sciatic nerve sections from MTMR2,13 knockout mice (Berger et al., 2011). The direct effect on phosphoinositide composition could alter the promyelinating signaling cascade and link the mutation to the demyelinating phenotype. [For a good review on ErB signaling and trafficking in CMT we refer the reader to Lee et al. (2017)].

Lastly, MTMR2 has been shown to interact with NEFL, indicating a common pathway could underlie the pathology causing CMT4B and CMT2E explaining the similar phenotypes observed (Previtali et al., 2003).

### CMT4C

CMT4C, also an autosomal recessive disorder with an early onset, is characterized by hypomyelination. This hypomyelination is caused by both, nonsense and missense mutations in the *SH3TC2* gene leading to a loss of function (Senderek et al., 2003). Over 20 different mutations have been reported to date. SH3TC2 is expressed in Schwann cells but not neurons of the PNS (Vijay et al., 2016). Although its exact molecular mechanism has not been identified, SH3TC2 has been implicated in the ErbB-neuregulin1 signaling axis, a crucial pathway for PNS myelination. Neuregulin1 binds ErB3, which activates ErbB2. This receptor complex is internalized for downstream promyelin-signaling (Di Guglielmo et al., 1994; Zastrow and Sorkin, 2007; Birchmeier and Bennett, 2016). It has been proposed that SH3TC2 plays a role in endocytosis, as ErbB2 internalization is reduced in SH3TC2 knockout Schwann cells. Moreover, overexpression of SH3TC2 increased internalization of ErbB2, co-immunoprecipitated with ErbB2 and is co-internalized with it upon stimulation (Gouttenoire et al., 2013). To date, the molecular role of SH3TC2 in endocytosis remains undetermined. CMT4C mutations of SH3TC2 impair the localization of SH3TC2 to the plasma membrane (Lupo et al., 2009) and impair ErbB2 uptake in Schwann cells (Gouttenoire et al., 2013). These findings indicate a role for SH3TC2 in receptor uptake and that endocytic dysfunctions in CMT mutants contribute to the pathology. However, many more roles of SH3TC2 at different stages of the endolysosomal pathway have arisen that may contribute to the pathology seen in CMT4C.

Besides localizing to the plasma membrane, SH3TC2 is found on recycling endosomes (the perinuclear recycling compartment in Schwann cells) that bind to active Rab11 (Arnaud et al., 2009; Roberts et al., 2010; Stendel et al., 2010). Interestingly, CMT4C mutants of SH3TC2 showed no interaction with Rab11 and no localization to recycling endosomes. In HeLa cells, mutant SH3TC2 has been shown to promote the recycling of the transferrin receptor (TfR) back to the plasma membrane, whereas transient expression of WT SH3TC2 decreased TfR recycling (Roberts et al., 2010). This suggests that SH3TC2 either acts as a competitor to TfR recycling or negatively regulates the recycling of TfR directly and that this is disrupted by the CMT4C mutations. Lastly, the SH3TC2 mutants show decreased myelin protein synthesis in Schwann cells and dominant negative Rab11 has been shown to lead to myelin defects *in vitro*, whereas constitutively active Rab11 increased myelination (Stendel et al., 2010). This indicates a direct role of Rab11 in the myelination process and therefore a likely role for its effector SH3TC2. Vijay et al. have identified Integrinα6 as a SH3TC2 associated protein in retinal pigment epithelial cells. Integrinα6 is a laminin receptor known to recycle via Rab11 endosomes involved in maintaining structural integrity of the myelin sheath (Vijay et al., 2016). It would be interesting to see if the recycling of ErB and Integrinα6 is also disrupted by the CMT4C mutations, as ErB2 depends on rapid recycling for proper signaling. Further, it remains to be dissected, whether the effects described for SH3TC2 affecting endocytosis and recycling are shared effects of the same pathway or separate pathways.

### CMT4D

CMT4D is a demyelinating, autosomal recessive type of the disorder caused by a mutation in N-myc downstream regulated 1 (NDRG1; Kalaydjieva et al., 2000). The most common truncation mutation (R148X) leads to a loss of function showing similar phenotypes as complete deletion of the protein (King et al., 2011). Its high and exclusive expression in Schwann cells in the PNS suggest a Schwann cell specific role, although the precise role remains to be determined (Okuda et al., 2004). In prostate cancer cell lines, NDRG1 was identified as a Rab4 effector protein. NDRG1 was shown to be involved in the fast recycling of TfR, as recycling was slowed down when NDRG1 was knocked down. Further, NDRG1 binds PI_4_P but is recruited to endosomes independently of its effector Rab4 (Kachhap et al., 2007). Yet overexpression of mutant NDRG1 with Rab4 in HeLa cells resulted in enlarged Rab4 endosomes compared to WT NDRG1 (Li et al., 2017). In cancer cell lines, NDRG1 is involved in recycling of e-cadherin (Kachhap et al., 2007). This leads to speculate that other recycling pathways specific to Schwann cell myelination and Schwann cell/axonal communication might be disrupted by loss of NDRG1 function. Recycling of both, ErbB and the low density lipoprotein (LDL) receptor are significantly reduced in CMT4D (Pietiäinen et al., 2013). Investigating this abnormality in LDL receptor recycling showed that NDRG1 has a role as a negative regulator of receptor degradation. The observation of decreased LDL receptor recycling led to the hypothesis that NDRG1 normally prevents ubiquitination of LDL receptor, leading to its recycling back to the plasma membrane. When NDRG1 is dysfunctional, LDL receptor is marked for degradation, therefore the receptor is not recycled back to the plasma membrane. This leads to a shortage of receptors available for endocytosis and thus a shortage of LDL (Pietiäinen et al., 2013). In addition, this experimental set up also showed a second effect of NDRG1 depletion: abnormal endosomal maturation. LDL receptors were found to be trapped in MVBs. These MVBs showed an increased number of ILVs (despite a downregulation of ESCRT proteins), yet were still positive for early endosomal marker EEA1, indicating disturbed endosomal maturation. This phenotype in combination with the observed slowed degradation of LDL receptor indicates a delayed fusion of MVBs with lysosomes. Prenylated Rab Acceptor 1 protein (PRA1) was identified as a NDRG1 interactor (King et al., 2011) and overexpression of PRA1 was able to partially rescue LDL receptor phenotype of NDRG1 depletion (Pietiäinen et al., 2013). PRA1 regulates several Rab GTPases including Rab4, Rab5, Rab7, and Rab9 (Bucci et al., 1999). Since Rabs are key players in intracellular trafficking, it is likely that dysregulation of PRA1 contributes to the pathomechanism of delayed lysosomal fusion. However, how PRA1 function is altered by the loss of function in *NDRG1* remains to be investigated.

Interestingly, NDRG1 showed interaction with ApoA1 and A2, both are proposed to be involved in Schwann cell lipid trafficking (Hunter et al., 2005). Whether a parallel recycling effect based on NDRG1s interaction with Rab4 is involved, how NDRG1 functions as a ubiquitin inhibitor and what mechanisms are altered by PRA1 activity in CMT4D are all questions that remain to be answered.

### CMT4H

Another autosomal recessive, early onset disorder is CMT4H caused by mutations in the *FGD4* gene encoding FYVE, RhoGEF and PH domain containing protein 4 (FGD4) (Delague et al., 2007). Over 20 mutations have been reported so far, many of which result in a truncated and loss of function mutation or missense mutations in the PI-recognition domains (two PH domains recognize PI_3_,_4_,_5_P_3_, PI_4_,_5_P_2_, and PI_3_,_4_P_2_, FYVE domain binds PI_3_P) (Argente-Escrig et al., 2019). FGD4 is a GEF for the Rho GTPase Cdc42 and Rac1 (Obaishi et al., 1998; Umikawa et al., 1999). As deletion of Cdc42 in adult Schwann cells of mice shows a similar phenotype to adult deletion of FGD4 and levels of active Cdc42 are reduced in sciatic nerves of adult FGD4 knockouts as well as in cultured Schwann cells (Horn et al., 2012), the Cdc42 pathway is probably disrupted in CMT4H. Even though CMT4H is an early onset disorder and FGD4 expression in Schwann cells is required for proper myelin development it is also important for myelin maintenance as an induced knockout in adult Schwann cells leads to myelin defects. Interestingly and tying FGD4 to the endocytic section of this review: depletion of endogenous FGD4 inhibits the internalization of TfR in rat Schwann cells (Horn et al., 2012). Unfortunately, the molecular mechanism of FGD4’s involvement in endocytosis is still unknown. However, Cdc42 has a proposed role in endocytosis, by enabling clathrin mediated endocytosis via actin polymerization (Yang et al., 2001; Bu et al., 2010). This observation opens up the possibility that other internalization processes i.e., ErbB are disrupted leading to the pathology.

When overexpressed in rat motoneurons or rat RT4 schwannoma cells, wild type FGD4 co-localized with f-actin in the growth cone and tips of neurites and increased the number of filopodia-like microspikes. Overexpression of truncated FGD4, still revealed colocalization with f-actin, but showed a reduced number of microspikes with altered curly morphology (Delague et al., 2007). This observation indicates that FGD4 plays a role in the structural organization of microfilaments during cellular growth, which is possibly disrupted by the loss of function mutations in CMT4H. Further, Cdc42 has been implicated in the reorganization of the cytoskeleton (Daub et al., 2001; Nakanishi and Takai, 2008; Li et al., 2021). However, no abnormalities in the organization of microfilaments or microtubules were observed in patient fibroblasts (Delague et al., 2007). It is possible that changes unnoticed in fibroblasts are detrimental for neurons or Schwann cells, considering their long and polarized structure.

### CMT4J

CMT4J is caused by a partial loss-of-function mutation on FIG4 phosphoinositide 5-phosphatase (FIG4), a ubiquitously expressed phosphoinositide 5 phosphatase, dephosphorylating PI_3_,_5_P_2_ to PI_3_P. CMT4J is a neuropathy with myelin defects and axonal loss in the periphery caused by a compound heterozygotic combination of a missense allele with a null allele. A loss of FIG4 function, counterintuitively, decreases the levels of PI_3_,_5_P_2_ (Chow et al., 2007), as FIG4 also activates PIKFYVE, a PI5K on endosomes (Rudge et al., 2004). The CMT mutant of FIG4 is not stabilized, leading to protein instability and reduced levels of FIG4 (Lenk et al., 2011). In FIG4 deficient cells, lysosomes and endosomes are enlarged, showing that a tight PI regulation is important for proper endosome maturation and following fusion with lysosomes. Interestingly, this phenotype can be rescued in drosophila by overexpression of an enzymatically inactive FIG4, indicating that its loss of function goes beyond its phosphatase activity (Bharadwaj et al., 2016). FIG4 also interacts with MTMR2 in neurons and Schwann cells, indicating a shared pathomechanism in CMT4B and CMT4J. As they both have an effect on PI_3_,_5_P_2_, in Schwann cells the loss of FIG4 function in MTMR2 null mice rescues the myelin outfolding phenotype. As the loss of MTMR2 leads to more PI_3_,_5_P_2_, the reduction of FIG4 and therefore the decrease in PIKFYVE reduces levels of PI_3_,_5_P_2_ (Vaccari et al., 2011). The overlap of these two CMT subtypes working on a shared pathway are a great opportunity to figure out the downstream changes of altered PI_3_,_5_P_2_ levels leading to the pathology and should be investigated in more detail. *In vivo*, conditional inactivation of FIG4 lead to axonal degeneration and Schwann cell demyelination, possibly by a defective transport of cholesterol (Vaccari et al., 2015). Further, altered levels of autophagic markers were observed in neurons and astrocytes of mice models of CMT4J. These altered levels indicate a decrease in autophagic flux, proposed to be due to defective fusion with lysosomes (Ferguson et al., 2009; Vaccari et al., 2015).

### CMT1X

CMT1X is caused by mutations in the *GJB1* gene located on the X chromosome encoding Gap junction protein beta 1 (GJB1) also known as Connexin32 (Cnx32) (Bergoffen et al., 1993). Over 400 mutations have been reported to cause CMT1X, with many of them being loss of function mutations, where WT GJB1 normally forms gap junctions in the peripheral nerve ensuring intercellular communication (Kleopa et al., 2012). Abnormal trafficking has been reported for CMT1X in two ways. First, mutant GJB1 is not properly trafficked anterogradely to the plasma membrane. Instead, mutant GJB1accumulates in the endoplasmic reticulum or Golgi leading to a lack of intercellular gap junctions (Deschênes et al., 1997; Yum et al., 2002; Abrams et al., 2003; Kleopa et al., 2006). However, some mutant forms of GJB1 form functional gap junctions indicating a second, parallel pathogenic mechanisms (Castro et al., 1999). Vavlitou et al. (2010) have shown that lack of GJB1 can cause defects in axonal retrograde transport and neurofilament abnormalities before onset of demyelination in mice. Neurofilaments were more densely packed and dephosphorylated in the absence of GJB1, probably leading to deficient axonal transport as indicated by the accumulation of dynein and other markers usually transported along the axon (Vavlitou et al., 2010). Although direct evidence for trafficking defects is missing this indicates a pathomechanism beyond the lack of gap junctions that requires further investigation as to how GJB1 affects neurofilaments and thus trafficking, especially if the trafficking of receptor tyrosine kinases is altered by the neurofilament abnormalities observed. A potentially common pathway could be the altered MEK-ERK signaling observed in GJB1 deficient Schwann cells leading to altered expression profiles of MEK-ERK regulated proteins (Groh et al., 2010).

With all studies targeting the experimental questions using specific approaches (e.g., looking at one receptor or one specific downstream protein related to the affected GEFs), it would be interesting to set up comparative studies between the CMT subtypes targeting potential shared proteins. This would help to reveal if there are common denominators affected.

## Demyelinating CMT

### CMT1A

Also prominently demyelinating subtypes of CMT have shown several transport deficits (Figure 2). CMT1A is the most common cause of CMT, an autosomal dominant type caused by a duplication of the gene encoding peripheral myelin protein 22 (PMP22), leading to overexpression and aggregation. Even though not the focus of many studies, intracellular transport is heavily involved in the processing of PMP22 in several aspects. For example, a prominent pathway in the degradation of misfolded proteins and aggregates is autophagy. With the observation of an increase in misfolded PMP22 and its aggregates, it is reasonable to speculate that autophagy plays an important role in protein clearance of CMT1A. Patient derived fibroblasts show increased levels of autophagic markers (Lee et al., 2018). Indeed, autophagy activation by rapamycin was shown to improve myelination in demyelinating CMT mouse DRG cultures (Rangaraju et al., 2010) and in mice (Nicks et al., 2014). Further, a recent study by Marinko et al. shows that the expression of PMP22 is negatively correlated to the trafficking of newly synthetized PMP22 to the plasma membrane. When too much PMP22 is expressed, the proteasomes of the ER’s membrane protein quality control become saturated as most of PMP22 is very unstable and needs to be degraded. Upon saturation of the proteasomes, misfolded PMP22 is accumulating and aggregating, leading to a decrease of functional PMP22 being trafficked to the plasma membrane, ultimately leading to demyelination (D’Urso et al., 1998; Marinko et al., 2020). Increasing autophagy can lessen the burden of proteasomes and thus increase PMP22 transport to the plasma membrane, showing how trafficking defects in Schwann cells as secondary mechanisms can lead to demyelination (Fortun et al., 2007; Rangaraju et al., 2010). Further, in patient nerves increased levels of ErbB2/3 were found in Schwann cells, this could potentially mean a defective degradation pathway (Massa et al., 2006). Whether the possible defect in degradation is a secondary effect due to a saturated autophagic system or a parallel defect remains to be investigated. Interestingly, early studies report axonal transport defects in mice with mutated PMP22 showing slowed axonal transport of neurofilaments, decreased microtubule stability and abnormal neurofilament phosphorylation opening up the possibility of secondary trafficking defects in demyelinated neurons (De Waegh and Brady, 1990; de Waegh et al., 1992).

### CMT1C

In autosomal dominant CMT1C point mutations in *LITAF*, also known as *SIMPLE*, are identified to cause the disease (Street et al., 2003). *LITAF* encodes a 161 amino acid protein (Lipopolysaccharide-induced tumor necrosis factor-alpha factor) with one transmembrane domain that is inserted to the membrane post-translationally, all mutations occur in this domain. Therefore, mutated LITAF no longer localizes to endosomes but is cytosolic (Lee et al., 2011). LITAF interacts with STAM1, Hrs and TSG101, ESCRT proteins of the degradative pathway (Lee et al., 2012). The cytosolic mutant competes with endosomally located LITAF for Hrs, leading to deficient Hrs recruitment to endosomes and a lack of ESCRT recruitment. This lack of recruitment has been shown to decrease EGFR degradation leading to prolonged ERK1/2 signaling. Late endosomes/lysosomes are enlarged in patients’ fibroblasts, similarly to the phenotype reported for mutant FIG4 in CMT4J showing a defect in the degradative pathway (Edgar et al., 2020). By activating cation channel TRPML1, a homeostasis regulator of lysosomes in mammalian cells, the authors were able to rescue the vacuolar phenotype of both LITAF or FIG4 knockout cells demonstrating a common pathway. It will be essential to unravel how the enlarged endolysosomes in both CMT types lead to the pathology. A possibility offers deficient ErbB3 degradation that has been shown in CMT mutants also leading to prolonged ERK1/2 signaling and demyelination (Lee et al., 2012).

An overall summary of how genes causing CMT affect trafficking in Schwann cells is shown in Figure 2.

## Treatments

Currently there is no cure for CMT, nor is there a treatment that addresses the specific phenotypes of CMT. Patients can only battle the disorder with physical and occupational therapies, as well as with pain-relief medication or surgeries to cope with the symptoms. Pinpointing the specific disruption in intracellular transport can help to identify new therapeutic targets, for current therapeutic prospects in CMT, we refer to two excellent recent reviews (Beijer et al., 2019; Morena et al., 2019). However, since axonal transport is disrupted in many subtypes of CMT, we briefly want to discuss the potential of HDAC6 inhibitors as a treatment for CMT.

HDAC6 inhibitor treatment alleviates many of the trafficking phenotypes in several models of different CMT types. HDAC6 deacetylates tubulin, making it unstable. By inhibiting HDAC6, tubulin remains more stable, which improves the basis for intracellular transport. Other targets of HDAC6’s deacteylation function include heat shock protein 90, cortactin and Miro1, linking it to the elimination process of misfolded proteins and mitochondrial transport. HDAC6 is also proposed to play a role in response to eliminate misfolded proteins independent of its deacetylation function. It shows a high binding affinity for ubiquitinated proteins and can interact with dynein motor proteins directly. Especially in different CMT2 types HDAC6 inhibitors have been shown to be beneficial (for a review please see Rossaert and Van Den Bosch, 2020). For example, treatment with HDAC6 inhibitor partially rescued phenotypes displayed by mice with mutant HSPB1, models for CMT2F (D’Ydewalle et al., 2011). But also in mice modeling CMT2D (caused by mutated GlyRS) the treatment with HDAC6 inhibitor has been shown to restore function (Benoy et al., 2018; Mo et al., 2018). Due to its direct interaction with Miro1 HDAC6 inhibitor was also tested as a treatment for CMT2A in a mouse model, where it even rescued motor dysfunction when given after symptom onset, giving it great therapeutic potential (Picci et al., 2020). Its role in misfolded protein clearance has shown a promising treatment strategy for CMT1A, where an HDAC6 inhibitor leads to improved nerve integrity as well as improved motor behavior in C22 mice (Ha et al., 2020). Overall, HDAC6’s inhibitors are shown to be promising therapeutic targets for several CMT subtypes that are currently being developed (Shen and Kozikowski, 2020). The beneficial effect of HDAC6 inhibitors on different CMT subtypes also shifts HDAC6’s involvement in the pathogenesis into focus of future research, also in the subtypes not yet explored. To develop new therapeutic strategies and model systems we refer to an excellent review by the Timmerman lab, highlighting recent advances in modeling CMT (Juneja et al., 2019).

## Concluding Remarks

Overall, it is fascinating to see that mutations in very diverse proteins can all lead to similar defects in intracellular transport. Whether the mutations cause a direct defect in traffic by affecting the stability of the cytoskeleton, or whether the trafficking defects are secondary due to disrupted signaling (which ultimately also affects the stability of the cytoskeleton) remains to be elucidated for many CMT subtypes. Here we tried to give enough detailed insights into the different trafficking defects observed to encourage more studies that investigate similar defects in multiple CMT disorders for better comparability in the search for a common denominator and to look at the system of intracellular transport as a highly fluid and dynamic system. The sheer number of results indicating trafficking defects in all the different CMT subtypes is a solid basis to suspect abnormal trafficking as the underlying pathomechanism. However, many studies that claim trafficking defects only look at static data and lack comparability with each other. More experiments generally looking at cytoskeletal integrity, mitochondrial transport, retrograde transport of receptors, recycling and degradation for each subtype are needed for a holistic approach to CMT pathology as well as, checking phenotypes reported for one subtype in the others. This also applies for CMT subtypes not mentioned in this review that have, as of yet, no or less reported trafficking phenotypes.

## Author Contributions

RM wrote the first draft of the manuscript and prepared the figures. KB and MB reviewed and edited the manuscript. All authors contributed to the article and approved the submitted version.

## Conflict of Interest

The authors declare that the research was conducted in the absence of any commercial or financial relationships that could be construed as a potential conflict of interest.

## Publisher’s Note

All claims expressed in this article are solely those of the authors and do not necessarily represent those of their affiliated organizations, or those of the publisher, the editors and the reviewers. Any product that may be evaluated in this article, or claim that may be made by its manufacturer, is not guaranteed or endorsed by the publisher.
